# 2-Chloro-*N*′-(5-hydr­oxy-2-nitro­benzyl­idene)benzohydrazide

**DOI:** 10.1107/S1600536810001303

**Published:** 2010-01-16

**Authors:** Cong-Shan Zhou, Tao Yang

**Affiliations:** a College of Chemistry and Chemical Engineering, Hunan Institute of Science and Technology, Yueyang, Hunan 414006, People’s Republic of China

## Abstract

The mol­ecule of the title Schiff base compound, C_14_H_10_ClN_3_O_4_, exists in a *trans* configuration with respect to the acyclic C=N bond. The dihedral angle between the two benzene rings is 62.37 (9)°. An intra­molecular C—H⋯O hydrogen bond is observed. In the crystal structure, adjacent mol­ecules are linked into a ribbon along [1
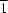
0] by O—H⋯O and N—H⋯O hydrogen bonds.

## Related literature

For the biological properties of Schiff bases, see: Mohamed *et al.* (2009 ▶); Ritter *et al.* (2009 ▶); Bagihalli *et al.* (2008 ▶). For related structures, see: Fun *et al.* (2008 ▶); Shafiq *et al.* (2009 ▶); Goh *et al.* (2010 ▶); Zhou *et al.* (2009 ▶); Zhou & Yang (2009 ▶).
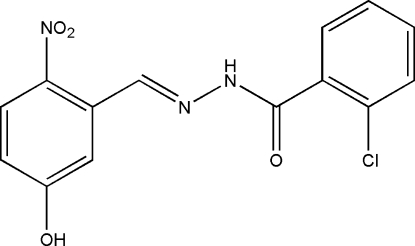


## Experimental

### 

#### Crystal data

C_14_H_10_ClN_3_O_4_
*M* *_r_* = 319.70
Triclinic, 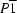

*a* = 7.2490 (2) Å
*b* = 9.4719 (3) Å
*c* = 10.4749 (4) Å
α = 100.623 (2)°
β = 97.433 (2)°
γ = 96.127 (2)°
*V* = 694.64 (4) Å^3^
*Z* = 2
Mo *K*α radiation
μ = 0.30 mm^−1^
*T* = 298 K
0.17 × 0.15 × 0.15 mm

#### Data collection

Bruker SMART 1000 CCD area-detector diffractometer
Absorption correction: multi-scan (*SADABS*; Sheldrick, 1996 ▶) *T*_min_ = 0.951, *T*_max_ = 0.957
4097 measured reflections
2900 independent reflections
2332 reflections with *I* > 2σ(*I*)
*R*_int_ = 0.016

#### Refinement

*R*[*F* ^2^ > 2σ(*F* ^2^)] = 0.037
*wR*(*F* ^2^) = 0.103
*S* = 1.03
2900 reflections
203 parameters
1 restraint
H atoms treated by a mixture of independent and constrained refinement
Δρ_max_ = 0.20 e Å^−3^
Δρ_min_ = −0.25 e Å^−3^

### 

Data collection: *SMART* (Bruker, 2007 ▶); cell refinement: *SAINT* (Bruker, 2007 ▶); data reduction: *SAINT*; program(s) used to solve structure: *SHELXTL* (Sheldrick, 2008 ▶); program(s) used to refine structure: *SHELXTL*; molecular graphics: *SHELXTL*; software used to prepare material for publication: *SHELXTL*.

## Supplementary Material

Crystal structure: contains datablocks global, I. DOI: 10.1107/S1600536810001303/ci5018sup1.cif

Structure factors: contains datablocks I. DOI: 10.1107/S1600536810001303/ci5018Isup2.hkl

Additional supplementary materials:  crystallographic information; 3D view; checkCIF report

## Figures and Tables

**Table 1:** Hydrogen-bond geometry (Å, °)

*D*—H⋯*A*	*D*—H	H⋯*A*	*D*⋯*A*	*D*—H⋯*A*
N2—H2⋯O2^i^	0.89 (1)	2.09 (1)	2.9591 (18)	165 (2)
O4—H4⋯O1^ii^	0.82	1.85	2.6708 (17)	176
C7—H7⋯O2	0.93	2.22	2.817 (2)	122

Symmetry codes: (i) 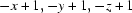; (ii) 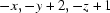.

**Fig. 1.**
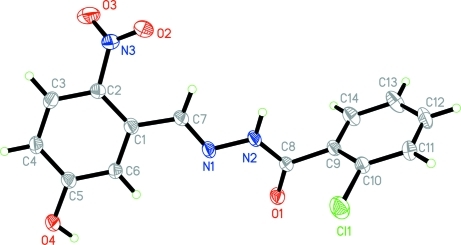
The molecular structure of the title compound, with displacement ellipsoids drawn at the 30% probability level.

**Fig. 2.**
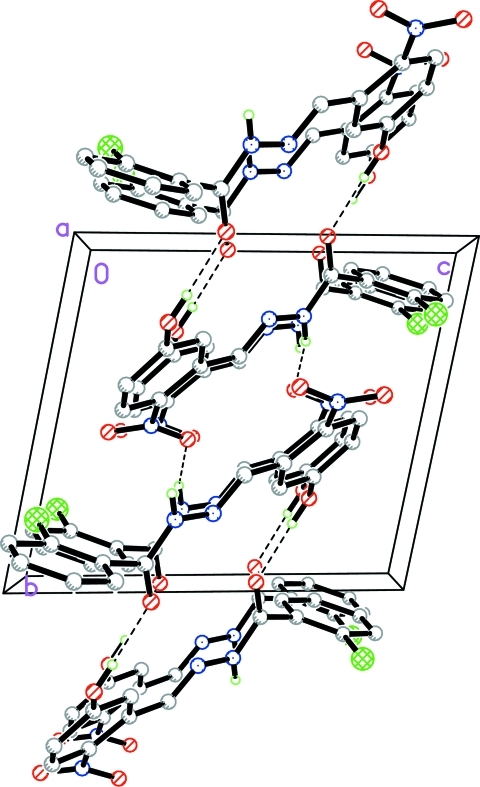
Part of the crystal packing of the title compound, viewed along the a axis. Hydrogen bonds are shown as dashed lines.

## supplementary crystallographic information

### Comment

Schiff bases usually possess excellent biological properties, such as antibacterial, antimicrobial, and antitumor (Mohamed *et al.*, 2009; Ritter *et al.*, 2009; Bagihalli *et al.*, 2008). Recently, a large number of Schiff bases derived from the reaction of aldehydes with benzohydrazides have been reported (Fun *et al.*, 2008; Shafiq *et al.*, 2009; Goh *et al.*, 2010). In this paper, the crystal structure of the title new Schiff base derived from the condensing of 5-hydroxy-2-nitrobenzaldehyde with 2-chlorobenzohydrazide in methanol is reported.

Bond lengths in the title molecule (Fig. 1) are comparable to those observed in related structures (Zhou *et al.*, 2009; Zhou & Yang, 2009). The molecule exists in a *trans* configuration with respect to the acyclic C═N bond. The dihedral angle between the two benzene rings is 62.37 (9)°. An intramolecular C—H···O hydrogen bond is observed.

In the crystal structure, intermolecular N—H···O and O—H···O hydrogen bonds link adjacent molecules into a ribbon along [110] (Table 1 and Fig. 2).

### Experimental

5-Hydroxy-2-nitrobenzaldehyde (1.0 mmol, 167.1 mg) and 2-chlorobenzohydrazide (1.0 mmol, 170.0 mg) were dissolved in a methanol solution (30 ml). The mixture was stirred for 30 min at room temperature. The resulting solution was left in air for a few days, yielding colourless block-shaped crystals.

### Refinement

Atom H2 was located in a difference map and refined with the N–H distance restrained to 0.90 (1) Å. The remaining H atoms were positioned geometrically [C–H = 0.93 Å and O–H = 0.82 Å] and refined using a riding model, with *U*_iso_(H) = 1.2*U*_eq_(C) and 1.5*U*_eq_(O).

### Crystal data

**Table:**

C_14_H_10_ClN_3_O_4_	*Z* = 2
*M**_r_* = 319.70	*F*(000) = 328
Triclinic, *P*1	*D*_x_ = 1.528 Mg m^−^^3^
Hall symbol: -P 1	Mo *K*α radiation, λ = 0.71073 Å
*a* = 7.2490 (2) Å	Cell parameters from 1937 reflections
*b* = 9.4719 (3) Å	θ = 2.6–28.4°
*c* = 10.4749 (4) Å	µ = 0.30 mm^−^^1^
α = 100.623 (2)°	*T* = 298 K
β = 97.433 (2)°	Block, colourless
γ = 96.127 (2)°	0.17 × 0.15 × 0.15 mm
*V* = 694.64 (4) Å^3^	

### Data collection

**Table:**

Bruker SMART 1000 CCD area-detector diffractometer	2900 independent reflections
Radiation source: fine-focus sealed tube	2332 reflections with *I* > 2σ(*I*)
graphite	*R*_int_ = 0.016
ω scans	θ_max_ = 27.0°, θ_min_ = 2.0°
Absorption correction: multi-scan (*SADABS*; Sheldrick, 1996)	*h* = −8→9
*T*_min_ = 0.951, *T*_max_ = 0.957	*k* = −11→12
4097 measured reflections	*l* = −11→13

### Refinement

**Table:**

Refinement on *F*^2^	Primary atom site location: structure-invariant direct methods
Least-squares matrix: full	Secondary atom site location: difference Fourier map
*R*[*F*^2^ > 2σ(*F*^2^)] = 0.037	Hydrogen site location: inferred from neighbouring sites
*wR*(*F*^2^) = 0.103	H atoms treated by a mixture of independent and constrained refinement
*S* = 1.03	*w* = 1/[σ^2^(*F*_o_^2^) + (0.0433*P*)^2^ + 0.2231*P*] where *P* = (*F*_o_^2^ + 2*F*_c_^2^)/3
2900 reflections	(Δ/σ)_max_ = 0.001
203 parameters	Δρ_max_ = 0.20 e Å^−^^3^
1 restraint	Δρ_min_ = −0.25 e Å^−^^3^

### Special details

**Table:**

Geometry. All esds (except the esd in the dihedral angle between two l.s. planes) are estimated using the full covariance matrix. The cell esds are taken into account individually in the estimation of esds in distances, angles and torsion angles; correlations between esds in cell parameters are only used when they are defined by crystal symmetry. An approximate (isotropic) treatment of cell esds is used for estimating esds involving l.s. planes.
Refinement. Refinement of F^2^ against ALL reflections. The weighted R-factor wR and goodness of fit S are based on F^2^, conventional R-factors R are based on F, with F set to zero for negative F^2^. The threshold expression of F^2^ > 2sigma(F^2^) is used only for calculating R-factors(gt) etc. and is not relevant to the choice of reflections for refinement. R-factors based on F^2^ are statistically about twice as large as those based on F, and R- factors based on ALL data will be even larger.

### Fractional atomic coordinates and isotropic or equivalent isotropic displacement parameters (Å^2^)

**Table:**

	*x*	*y*	*z*	*U*_iso_*/*U*_eq_	
Cl1	0.21511 (7)	0.78685 (7)	0.05703 (5)	0.06308 (19)	
N1	0.2273 (2)	0.77176 (15)	0.47983 (14)	0.0378 (3)	
N2	0.3525 (2)	0.78387 (15)	0.39232 (15)	0.0387 (3)	
N3	0.3042 (2)	0.46714 (16)	0.72546 (17)	0.0469 (4)	
O1	0.30056 (19)	1.01035 (13)	0.37374 (13)	0.0487 (3)	
O2	0.3739 (2)	0.42683 (16)	0.62639 (16)	0.0618 (4)	
O3	0.3681 (2)	0.44819 (19)	0.83292 (17)	0.0740 (5)	
O4	−0.33418 (18)	0.74516 (14)	0.71890 (14)	0.0501 (3)	
H4	−0.3206	0.8188	0.6885	0.075*	
C1	0.1112 (2)	0.63648 (17)	0.62866 (16)	0.0356 (4)	
C2	0.1367 (2)	0.54064 (17)	0.71438 (17)	0.0370 (4)	
C3	0.0078 (3)	0.51237 (18)	0.79603 (18)	0.0427 (4)	
H3	0.0290	0.4480	0.8518	0.051*	
C4	−0.1514 (3)	0.57918 (19)	0.79491 (18)	0.0418 (4)	
H4A	−0.2407	0.5575	0.8471	0.050*	
C5	−0.1775 (2)	0.67960 (18)	0.71488 (17)	0.0381 (4)	
C6	−0.0480 (2)	0.70698 (18)	0.63260 (17)	0.0372 (4)	
H6	−0.0679	0.7737	0.5790	0.045*	
C7	0.2412 (2)	0.66589 (18)	0.53720 (18)	0.0396 (4)	
H7	0.3336	0.6068	0.5214	0.047*	
C8	0.3786 (2)	0.90324 (17)	0.34225 (16)	0.0342 (4)	
C9	0.5138 (2)	0.89383 (17)	0.24509 (17)	0.0352 (4)	
C10	0.4520 (2)	0.84446 (18)	0.11239 (18)	0.0391 (4)	
C11	0.5744 (3)	0.8381 (2)	0.02143 (19)	0.0480 (5)	
H11	0.5302	0.8060	−0.0677	0.058*	
C12	0.7634 (3)	0.8801 (2)	0.0653 (2)	0.0551 (5)	
H12	0.8473	0.8770	0.0051	0.066*	
C13	0.8291 (3)	0.9268 (2)	0.1973 (2)	0.0598 (6)	
H13	0.9571	0.9537	0.2260	0.072*	
C14	0.7048 (3)	0.9337 (2)	0.2875 (2)	0.0497 (5)	
H14	0.7495	0.9651	0.3767	0.060*	
H2	0.421 (3)	0.713 (2)	0.373 (2)	0.080*	

### Atomic displacement parameters (Å^2^)

**Table:**

	*U*^11^	*U*^22^	*U*^33^	*U*^12^	*U*^13^	*U*^23^
Cl1	0.0402 (3)	0.0935 (4)	0.0521 (3)	0.0016 (2)	0.0072 (2)	0.0100 (3)
N1	0.0386 (8)	0.0387 (7)	0.0418 (8)	0.0101 (6)	0.0200 (6)	0.0102 (6)
N2	0.0396 (8)	0.0373 (7)	0.0475 (8)	0.0143 (6)	0.0243 (7)	0.0128 (6)
N3	0.0455 (9)	0.0406 (8)	0.0619 (11)	0.0148 (7)	0.0141 (8)	0.0196 (7)
O1	0.0609 (8)	0.0432 (7)	0.0561 (8)	0.0249 (6)	0.0327 (7)	0.0191 (6)
O2	0.0643 (10)	0.0625 (9)	0.0756 (10)	0.0356 (7)	0.0338 (8)	0.0261 (8)
O3	0.0729 (11)	0.0925 (12)	0.0715 (11)	0.0404 (9)	0.0110 (9)	0.0382 (9)
O4	0.0463 (7)	0.0567 (8)	0.0628 (9)	0.0224 (6)	0.0302 (6)	0.0280 (7)
C1	0.0368 (9)	0.0324 (8)	0.0399 (9)	0.0056 (6)	0.0127 (7)	0.0077 (7)
C2	0.0363 (9)	0.0340 (8)	0.0438 (9)	0.0088 (7)	0.0106 (7)	0.0103 (7)
C3	0.0490 (10)	0.0395 (9)	0.0454 (10)	0.0083 (8)	0.0141 (8)	0.0176 (8)
C4	0.0428 (10)	0.0447 (9)	0.0441 (10)	0.0073 (7)	0.0198 (8)	0.0149 (8)
C5	0.0382 (9)	0.0376 (8)	0.0419 (9)	0.0089 (7)	0.0143 (7)	0.0086 (7)
C6	0.0394 (9)	0.0371 (8)	0.0411 (9)	0.0100 (7)	0.0154 (7)	0.0138 (7)
C7	0.0382 (9)	0.0384 (9)	0.0489 (10)	0.0135 (7)	0.0182 (8)	0.0134 (8)
C8	0.0334 (8)	0.0372 (8)	0.0349 (9)	0.0093 (7)	0.0112 (7)	0.0078 (7)
C9	0.0366 (9)	0.0319 (8)	0.0410 (9)	0.0083 (6)	0.0163 (7)	0.0079 (7)
C10	0.0363 (9)	0.0426 (9)	0.0426 (10)	0.0083 (7)	0.0141 (7)	0.0124 (7)
C11	0.0512 (11)	0.0585 (11)	0.0399 (10)	0.0129 (9)	0.0199 (9)	0.0123 (8)
C12	0.0499 (12)	0.0622 (12)	0.0601 (13)	0.0098 (9)	0.0338 (10)	0.0110 (10)
C13	0.0373 (10)	0.0655 (13)	0.0723 (15)	−0.0002 (9)	0.0225 (10)	−0.0031 (11)
C14	0.0407 (10)	0.0555 (11)	0.0484 (11)	0.0057 (8)	0.0121 (8)	−0.0047 (9)

### Geometric parameters (Å, °)

**Table:**

Cl1—C10	1.7346 (18)	C4—C5	1.391 (2)
N1—C7	1.267 (2)	C4—H4A	0.93
N1—N2	1.3814 (18)	C5—C6	1.390 (2)
N2—C8	1.337 (2)	C6—H6	0.93
N2—H2	0.891 (10)	C7—H7	0.93
N3—O3	1.213 (2)	C8—C9	1.500 (2)
N3—O2	1.228 (2)	C9—C10	1.381 (2)
N3—C2	1.465 (2)	C9—C14	1.387 (3)
O1—C8	1.2268 (19)	C10—C11	1.381 (2)
O4—C5	1.353 (2)	C11—C12	1.378 (3)
O4—H4	0.82	C11—H11	0.93
C1—C6	1.395 (2)	C12—C13	1.376 (3)
C1—C2	1.399 (2)	C12—H12	0.93
C1—C7	1.470 (2)	C13—C14	1.385 (3)
C2—C3	1.384 (2)	C13—H13	0.93
C3—C4	1.374 (2)	C14—H14	0.93
C3—H3	0.93		
			
C7—N1—N2	114.29 (13)	C1—C6—H6	119.3
C8—N2—N1	121.18 (13)	N1—C7—C1	120.03 (14)
C8—N2—H2	119.8 (16)	N1—C7—H7	120.0
N1—N2—H2	119.0 (16)	C1—C7—H7	120.0
O3—N3—O2	122.61 (16)	O1—C8—N2	123.19 (14)
O3—N3—C2	118.25 (16)	O1—C8—C9	123.39 (14)
O2—N3—C2	119.14 (16)	N2—C8—C9	113.42 (13)
C5—O4—H4	109.5	C10—C9—C14	118.64 (15)
C6—C1—C2	116.75 (14)	C10—C9—C8	121.08 (15)
C6—C1—C7	119.39 (14)	C14—C9—C8	120.28 (16)
C2—C1—C7	123.86 (15)	C9—C10—C11	121.74 (17)
C3—C2—C1	122.08 (15)	C9—C10—Cl1	119.70 (12)
C3—C2—N3	115.99 (15)	C11—C10—Cl1	118.56 (15)
C1—C2—N3	121.91 (15)	C12—C11—C10	118.78 (18)
C4—C3—C2	120.15 (15)	C12—C11—H11	120.6
C4—C3—H3	119.9	C10—C11—H11	120.6
C2—C3—H3	119.9	C13—C12—C11	120.62 (17)
C3—C4—C5	119.29 (15)	C13—C12—H12	119.7
C3—C4—H4A	120.4	C11—C12—H12	119.7
C5—C4—H4A	120.4	C12—C13—C14	120.08 (19)
O4—C5—C6	122.39 (15)	C12—C13—H13	120.0
O4—C5—C4	117.36 (15)	C14—C13—H13	120.0
C6—C5—C4	120.23 (15)	C13—C14—C9	120.11 (19)
C5—C6—C1	121.40 (15)	C13—C14—H14	119.9
C5—C6—H6	119.3	C9—C14—H14	119.9

### Hydrogen-bond geometry (Å, °)

**Table:**

*D*—H···*A*	*D*—H	H···*A*	*D*···*A*	*D*—H···*A*
N2—H2···O2^i^	0.89 (1)	2.09 (1)	2.9591 (18)	165 (2)
O4—H4···O1^ii^	0.82	1.85	2.6708 (17)	176
C7—H7···O2	0.93	2.22	2.817 (2)	122

Symmetry codes: (i) −*x*+1, −*y*+1, −*z*+1; (ii) −*x*, −*y*+2, −*z*+1.
